# The effects of Tai Chi on physical function and safety in patients with rheumatoid arthritis: A systematic review and meta-analysis

**DOI:** 10.3389/fphys.2023.1079841

**Published:** 2023-01-26

**Authors:** Haiyang Wu, Qiang Wang, Guowei Wen, Junhao Wu, Yiru Wang

**Affiliations:** ^1^ Huangpu Branch, Shanghai Ninth People’s Hospital, Shanghai Jiao Tong University School of Medicine, Shanghai, China; ^2^ Shanghai Traditional Chinese Medicine (TCM)—Integrated Hospital, Shanghai University of Traditional Chinese Medicine, Shanghai, China; ^3^ Longhua Hospital, Shanghai University of Traditional Chinese Medicine, Shanghai, China

**Keywords:** physical exercise, arthritis, pain, joint tenderness, swollen joints, health assessment questionnaire

## Abstract

**Background:** Rheumatoid arthritis (RA) is a chronic, systemic, inflammatory autoimmune disease that results in the destruction of joints, connective tissues, muscle, tendons and fibrous tissue. Until now, there are no cure therapies.

**Objective:** We aimed to assess the effectiveness of Tai Chi (TC) on RA patients by meta-analysis.

**Methods:** The PubMed, Cochrane Library, EMBASE, web of science, China National Knowledge Infrastructure and Google Scholar were searched up to January 2023. We included randomized controlled trials (RCTs) or controlled clinical trials (CCTs) comparing TC to control conditions for RA patients. Review Manager (Version 5.3) software was used to analyze outcomes of time to walk 50 feet, joint tenderness, number of swollen joints or tender joints, handgrip strength, pain, the Health Assessment Questionnaire (HAQ) and withdraws overall.

**Results:** A total of 351 patients with RA from six RCTs and three CCTs were included for meta-analysis. TC could also significantly decrease withdrawals overall in studies (OR = 0.28, 95% CI 0.12 to 0.67, *p* = 0.002). No significant treatment effects of physical function were identified of the other outcomes.

**Conclusion:** Our findings indicated that TC was safe to RA patients, but it cannot improve physical function and pain. However, there is still lack of more evidence.

**Systematic Review Registration:** [https://www.crd.york.ac.uk/PROSPERO/display_record.php?RecordID=367498], identifier [CRD42022367498].

## Introdution

Rheumatoid arthritis (RA) is a prevalent disease with incidence by 8.2% (Finckh et al., 2022). RA presents a systemic inflammatory autoimmune disease that destroys the joints, connective tissues, muscle, tendons and fibrous tissue. The accurate aetiology of RA is still ambiguous, but it is well known that the development of RA is associated with genetic susceptibility, environmental factors and immune response (Scherer et al., 2020; Testa et al., 2021). RA is often progressive and primarily involves the pain, stiffness and swelling of joints (Han et al., 2004). Some extra-articular manifestations also usually happen, such as cardiovascular disease, respiratory disease, central and peripheral nervous system (Figus et al., 2021). When compared to the general population, those with RA have a 50% greater risk of cardiovascular death (Finckh et al., 2022). RA brings a substantial burden for both the individual and society, because of decline in physical function, quality of life, work capacity and societal participation, and major direct medical costs (Hsieh et al., 2020). Current therapeutic approaches for RA includes pharmacological and non-pharmacological approaches. Pharmacological methods refer to disease-modifying antirheumatic drugs, non-steroidal anti-inflammatory drugs, glucocorticoids and biological drugs (Fraenkel et al., 2021). Regarding non-pharmacological approaches, such as exercise, education, psychological and self-management therapies for RA patients were found to be beneficial in improving non-inflammatory symptoms (mainly functional disability, pain and fatigue) (Roodenrijs et al., 2021). However, no cure is currently available for RA (Nagy et al., 2022).

Recently, several clinical studies and systematic reviews suggested that physical activity attenuates inflammation, cardiovascular risk, psychological health and sleep in RA patients (Metsios et al., 2015; McKenna et al., 2017; Pope, 2020). As a mitigatory therapeutic exercise, Tai Chi (TC) has been practiced for centuries as a martial art in China. At the same time, it has been drawn more and more attention. After introduced to Europe and America, the viewpoints of TC shifted and it is nowadays well-known as a kind of exercise to treat patients with knee osteoarthritis (Wang et al., 2016). TC consists of a series of slow and purposeful movements that involve turning, shifting one’s weight from one leg to the other one, bending and unbending the legs with various arm movement, which is benefit for balance, flexibility, strength and function of human beings (Wu et al., 2004).

In RA, TC appears safe (Christie and Fongen, 2005) and improves pain and functional status of RA (Kirsteins et al., 1991; Wang et al., 2005; Wang, 2008). A review in year of 2004 by Han (Han et al., 2004) suggests that TC is beneficial on lower extremity range of motion for RA patients. However, in Han’s review the three included studies were only up to December 2003. Another review in year of 2019 by Mudano (Mudano et al., 2019) showed that it was uncertain whether TC had any effect on joint pain, activity limitation or function in RA, and important effects cannot be confirmed or excluded since all outcomes had very low-quality evidence. Nevertheless, an overview of systematic reviews suggests that clinical improvement of TC is achieved, although not statistically significant with regard to pain and disease pattern (Imoto et al., 2021). Additionally, a clinical study published in 2020 is not included in any systematic reviews or meta-analysis (Liang, 2020). Thus, the effectiveness of TC for RA is still considered unproven, because of lack of enough convincing evidence. Therefore, the aim of this study was to conduct a systematic review and meta-analysis for exploring effectiveness of TC and summarizing the existing literature.

## Materials and methods

The work was reported in line with PRISMA (Preferred Reporting Items for Systematic Reviews and Meta-Analyses) (Page et al., 2021) and registered in PROSPERO (registration identification: CRD42022367498; website: https://www.crd.york.ac.uk/PROSPERO/display_record.php?RecordID=367498).

### Search strategy

The search strategy was made by two reviewers (HYW and QW). They searched the following electronic databases (up to January 2023): PubMed, Cochrane Library, EMBASE, web of science, China National Knowledge Infrastructure and Google Scholar. The search strategy included “Tai Chi,” “Tai-Chi Chuan”, “Taiji” and “rheumatoid arthritis”. HYW manually screened conference proceedings (such as the International League of Associations for Rheumatology, the Chinese Rheumatology Association, and Chinese Journal of Rheumatology) and files from our department as supplemental material. Details of the English search strategy were shown in the Supplementary Appendix S1.

### Inclusion criteria

All studies searched were imported into Endnote X9. Firstly, two reviewers (HYW and QW) screened the titles and abstracts relevant to TC for patients suffering from RA independently. Then still independently these two reviewers read full articles and identified whether the study to be included or not according to the following inclusion criteria. Disagreements were solved by JHW. All the reviewers were trained together to fully understand the inclusion criteria, exclusion criteria and using method of Endnote software before starting selection.

#### Participants

Participants were adults (16 years of age and older) suffering from RA. Patients were diagnosed by rheumatologists or clinicians in the department of rheumatology.

#### Intervention and comparison

The eligible trials should be TC therapy which compared with no therapy, usual care, sham therapy or any active treatment. Different types of TC protocol and co-interventions were allowed. Additionally, there were no limitations of the frequency of TC exercise, time of every intervention or the duration of trials.

#### Outcomes

1 Main outcomes (physical function): Time to walk 50 feet, joint tenderness, number of swollen joints or tender joints, handgrip strength, pain and HAQ.
2 Additional outcome (safety): Withdrawals overall.

#### Study design

Randomized controlled trials (RCTs) and controlled clinical trials (CCTs) were considered whether published or not in this review. Studies were included without language limitations.

### Risk of bias and quality assessment

The risk of bias was assessed using Review Manager software (Version 5.3.5, The Nordic Cochrane Centre, Copenhagen; available from: http://community.cochrane.org) and the 2011 revised Guidelines and Handbooks for Systematic Reviews in the Cochrane Back Review Group (Cumpston, 2011) by two reviewers (HYW and GWW). This handbook recommended seven quality criteria, each of which was rated with yes, no or unclear. Details of seven quality criteria were as follows: Random sequence generation (selection bias), allocation concealment (selection bias), blinding of participants and personnel (performance bias), blinding of outcome assessment (detection bias), incomplete outcome data (attrition bias), selective reporting (reporting bias) and other bias. Disagreements were solved by a third party (YRW). A study would not be excluded even with a high risk, but it might degrade our confidence to recommend this cure strategy.

### Data extraction and meta-analysis

Two reviewers (HYW and QW) extracted data from the included studies independently by a pre-pilot standardized form, which included first authors’ last names, publication years, types of studies, characteristics of interventions and participants (included TC and comparison groups), outcome measures of effectiveness (efficacy of functional and clinical outcomes) and safety (withdrawals overall), methodological qualities, allocation concealments and durations of studies. Disagreements were solved by a third investigator (JHW) with discussion.

The extracted data were divided into two parts: characteristics of studies were shown in a table, outcome measures of effectiveness and side effects were imported into the Review Manager software for performing meta-analysis. The outcomes of effectiveness data in the TC and control groups were used to estimate the mean difference (MD) and 95% confidence intervals (CIs). The outcomes of safety data were in terms of odds ratio (OR). All reported values were two sided and *p* < 0.05 was considered to be statistically significant. All the data was performed on the Review Manager software by one reviewer (HYW).

Regarding the methodological (methodology of included studies) and clinical (clinical characteristics of the participants) heterogeneity, we evaluated as not homogeneous due to different intervention periods and various countries of subjects. Based on these, random-effect model was used to perform the analysis.

## Results

### Study selection

**FIGURE 1:**
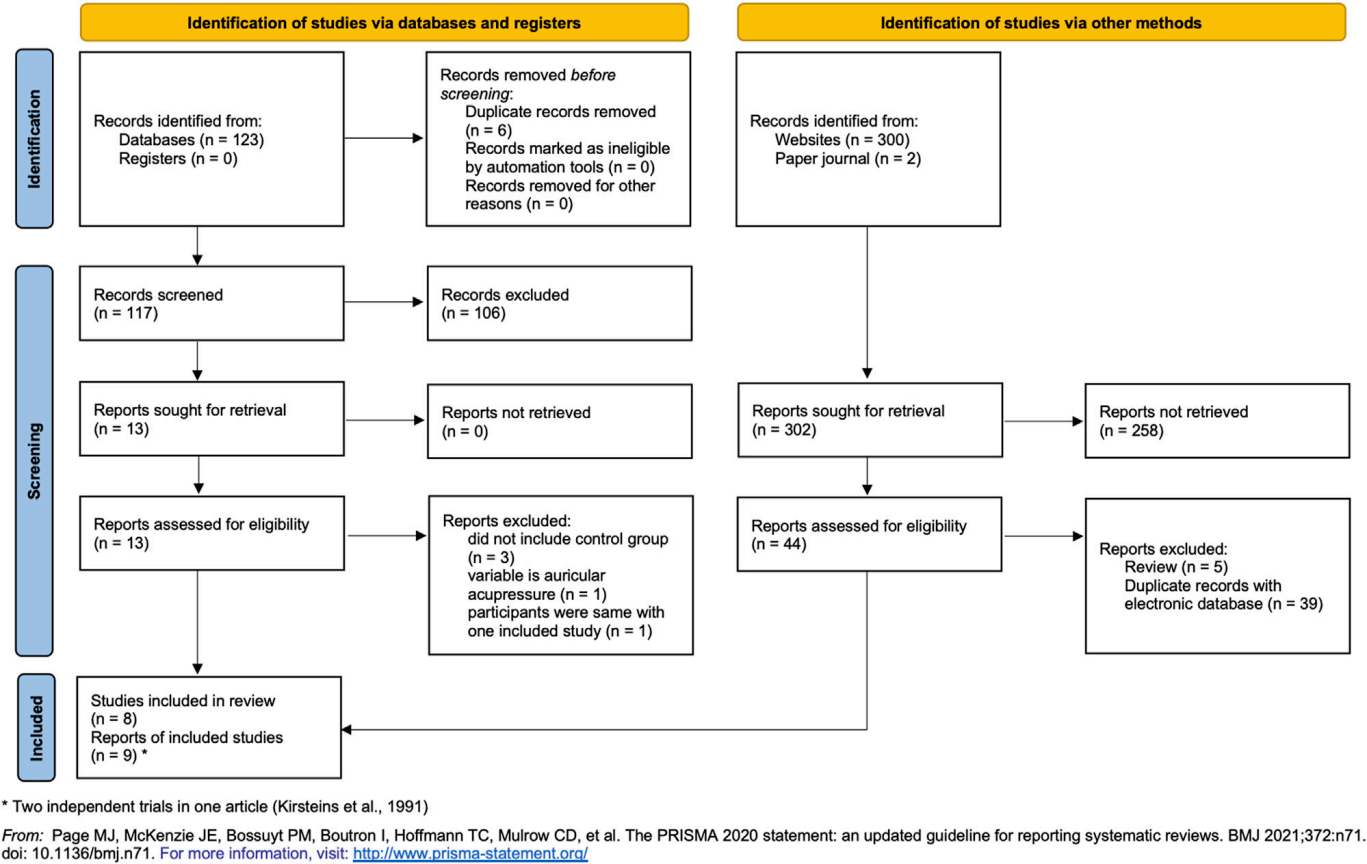
Flowchart of trial selection process. * Two trials in one article (Kirsteins et al., 1991).

After searching the electronic databases, websites (Google Scholar) and paper sources, we collected 425 articles. However, in the electronic databases 106 articles were excluded based on titles and abstracts after duplicates removed, only 13 records were screened by reading full texts. Among these, three studies did not include control group (Uhlig et al., 2005; Uhlig et al., 2010; Waite-Jones et al., 2013), the variable is auricular acupressure in one study (Lee et al., 2012), and participants were same in one study (Wang et al., 2005) with another included study (Wang, 2008). Regarding the websites results, the first three hundred records were evaluated, but there were no studies that could be included. In addition, two studies were found in paper journals, but did not meet the inclusion criteria. Finally, as two independent CCTs in the same article (Kirsteins et al., 1991), nine trials from eight articles included were analyzed (Van Deusen and Harlowe, 1987; Kirsteins et al., 1991; Zhu et al., 1999; Lee, 2005; Lee and Jeong, 2006; Wang, 2008; Shin et al., 2015; Liang, 2020). The difference lies in the frequency of TC intervention (details in Figure 1).

### Description of studies

The recruited articles were published from 1987 to 2020 years. The sample size ranged from 20 (Wang, 2008; Liang, 2020) to 68 (Zhu et al., 1999). All studies were single-center studies, while only one study was a multicenter one (Kirsteins et al., 1991). 351 RA participants were analyzed in this review. All patients satisfied the American College of Rheumatology 1987 revised classification criteria for RA. The frequency of TC was twice weekly (Kirsteins et al., 1991; Wang, 2008), once a week (Van Deusen and Harlowe, 1987; Kirsteins et al., 1991; Lee, 2005; Lee and Jeong, 2006; Shin et al., 2015) or once a day (Zhu et al., 1999; Liang, 2020). The duration of TC was 6 weeks (Lee, 2005), 8 weeks (Van Deusen and Harlowe, 1987; Zhu et al., 1999), 10 weeks (Kirsteins et al., 1991) and 12 weeks (Wang, 2008; Shin et al., 2015; Liang, 2020). The control groups were adopted usual activities without TC, advice about lifestyle, rest at home or oral the same medicine of TC group. The time to walk 50 feet was described in three studies (Kirsteins et al., 1991; Wang, 2008), joint tenderness in three studies (Kirsteins et al., 1991; Wang, 2008), the number of swollen joints in four studies (Kirsteins et al., 1991; Wang, 2008; Shin et al., 2015; Liang, 2020), the number of tender joints in two studies (Wang, 2008; Shin et al., 2015), handgrip strength in three studies (Kirsteins et al., 1991; Wang, 2008), pain in three studies (Lee, 2005; Lee and Jeong, 2006; Wang, 2008), HAQ in two studies (Wang, 2008; Shin et al., 2015; Liang, 2020), withdrawals overall during the study (Van Deusen and Harlowe, 1987; Kirsteins et al., 1991; Zhu et al., 1999). No studies described patients’ cost (details in Table 1).

**TABLE 1:** Characteristics of included studies.

Author	Design	Participants	Interventions	Comparison	Outcomes
Kirsteins 1991-1	CCT	47 adults (age 37–70 years, 42 females and 5 males) with RA. 25 patients in TC group and 22 in control group	Series of 15 movements extracted from Yang Style TC	Usual activities without TC	Joint tenderness, written functional, number of swollen joints, time to walk 50 feet, handgrip strength, safety
		Inclusion criteria: ambulatory adults with RA after age 18 and on a stable regimen of medications for a sufficient time for maximal results	Frequency: Once per week for 10 weeks, for 60 min sessions		
Kirsteins 1991-1	CCT	28 adults (age 38–72 years, 21 females and 7 males) with RA. 18 patients in TC group and 10 in control group	Series of 15 movements extracted from Yang Style TC	Usual activities without TC	Joint tenderness, written functional, number of swollen joints, time to walk 50 feet, handgrip strength, safety
		Inclusion criteria: Ambulatory adults with RA after age 18 and on a stable regimen of medications for a sufficient time for maximal results	Frequency: Twice per week for 10 weeks, for 60 min sessions		
Lee 2005	RCT	31 adults (age >30 years, all females) with RA. 16 patients in TC group and 15 in control group	Frequency: Once per week for 6 weeks, for 60 min sessions	Usual activities without TC	Pain (VAS) Mood (Profile of Mood State)
		Inclusion criteria: diagnosed RA in Dong-A University			Fatigue
Lee 2006	CCT	61 adults (All married females) with RA. 32 patients in TC group and 29 in control group	Frequency: Once per week for 12 weeks, for 50 min sessions	Usual activities without TC	Pain (VAS)
		Inclusion criteria: diagnosed RA in Dong-A University, no movement restrictions			Fatigue
Liang 2020	RCT	20 adults (age 30–65 years, 16 females and 4 males) with RA. 10 patients in TC group and 10 in control group	Frequency: Once everyday for 12 weeks, for 50 min sessions	Usual oral medicine treatment	HAQ, ESR, and CRP, number of swollen joints
		Inclusion criteria: Diagnosed RA according to 2010 ACR criteria			
Shin 2015	RCT	43 adults (age>50 years) with RA. 29 patients in TC group and 14 in control group	Twelve Movement TC	Received information about lifestyle modification and advice about appropriate regular exercises	Number of swollen joints and tender joints, HAQ, ESR, and CRP
		Inclusion criteria: more than 50 years old, sedentary lifestyle (no participation in structured exercise for the preceding 6 months), and stable disease (no changes in disease-modifying anti-rheumatic drugs or steroid in the last 3 months)	Frequency: Once per week for 3 months, for 60 min sessions		
Van Deusen 1987	RCT	33 adults (age 29–80 years) with RA. 17 patients in TC group and 16 in control group	TC ROM Dance program (including health education)	Rested at home, received a brochure which explained the program but no specific instructions	Shoulder flexion, shoulder internal and external rotation, wrist extension and flexion, ankle plantar flexion, lower extremity flexion, safety
		Inclusion criteria: ambulatory adults with RA who had medical recommendations for home rest and exercise and no prior ROM Dance experience	Frequency: Once per week for 8 weeks, for 90 min sessions		
Wang 2008	RCT	20 adults (age > 18 years) with RA. 10 patients in TC group and 10 in control group	Yang style TC	Usual physical activities, but not to participate in additional strength training other than class stretching exercises	ACR 20 response criterion, functional capacity, health-related quality of life and depression index
		Inclusion criteria: adults with functional class I or II RA (ACR criteria)	Frequency: Twice per week for 12 weeks, for 60 min sessions		
Zhu 1999	RCT	68 adults (age 16–56 years) with RA. 35 patients in TC group and 33 in control group	Oral San Bi recipe and exercise (slow running, walk, gymnastics and TC)	oral San Bi recipe in the same way but no exercise	safety
		Inclusion criteria: adults diagnosed with RA (ACR criteria)	Frequency: Once a day for 2 months, for 60 min sessions		

CCT, non-randomized controlled clinical trial; yrs, years; RA, rheumatoid arthritis; TC, Tai Chi; RCT, randomized controlled trial.

### Risk of bias and quality

**FIGURE 2:**
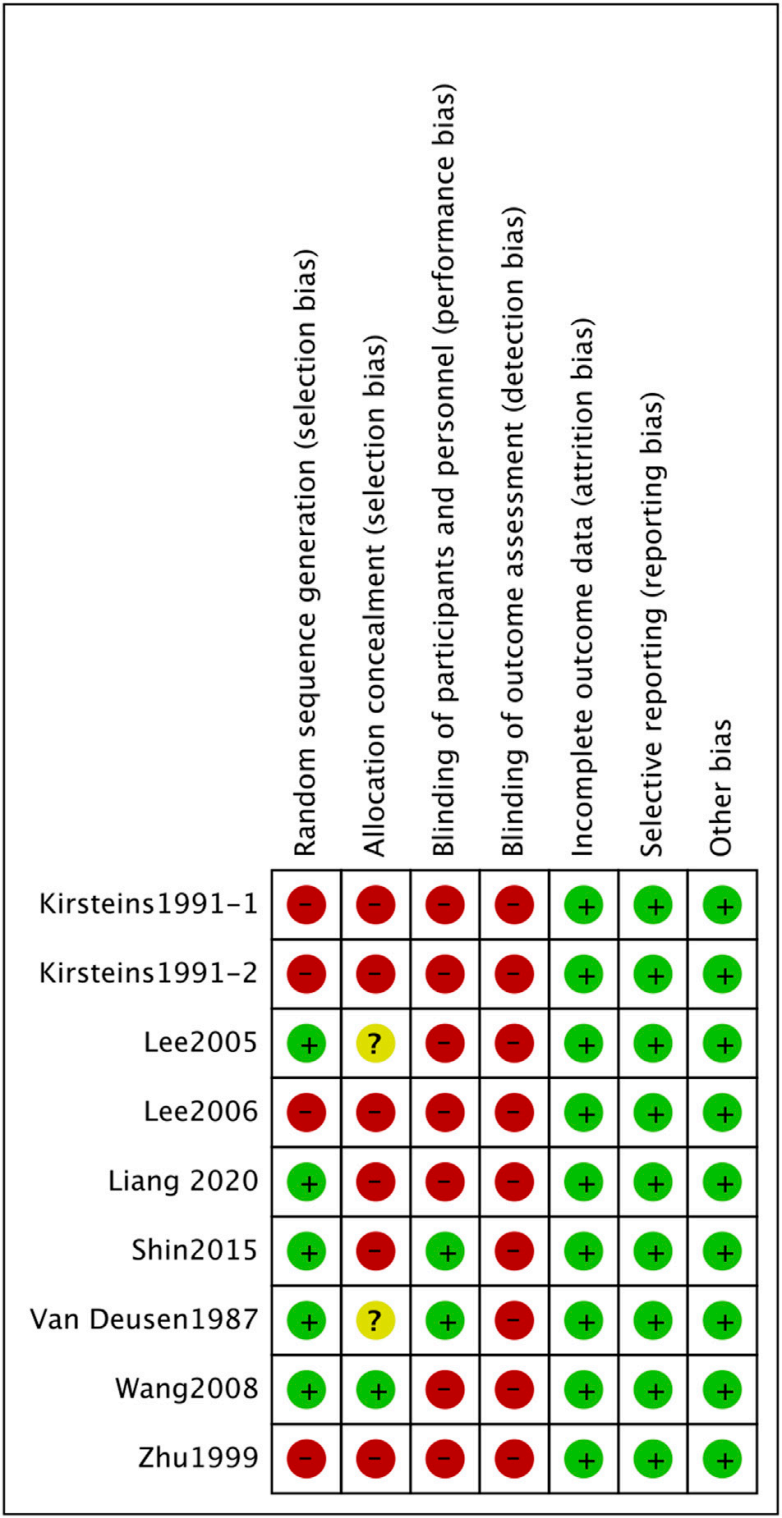
Risk of bias graph. Review authors’ judgements about each risk of bias item presented as percentages across all included studies.

**FIGURE 3:**
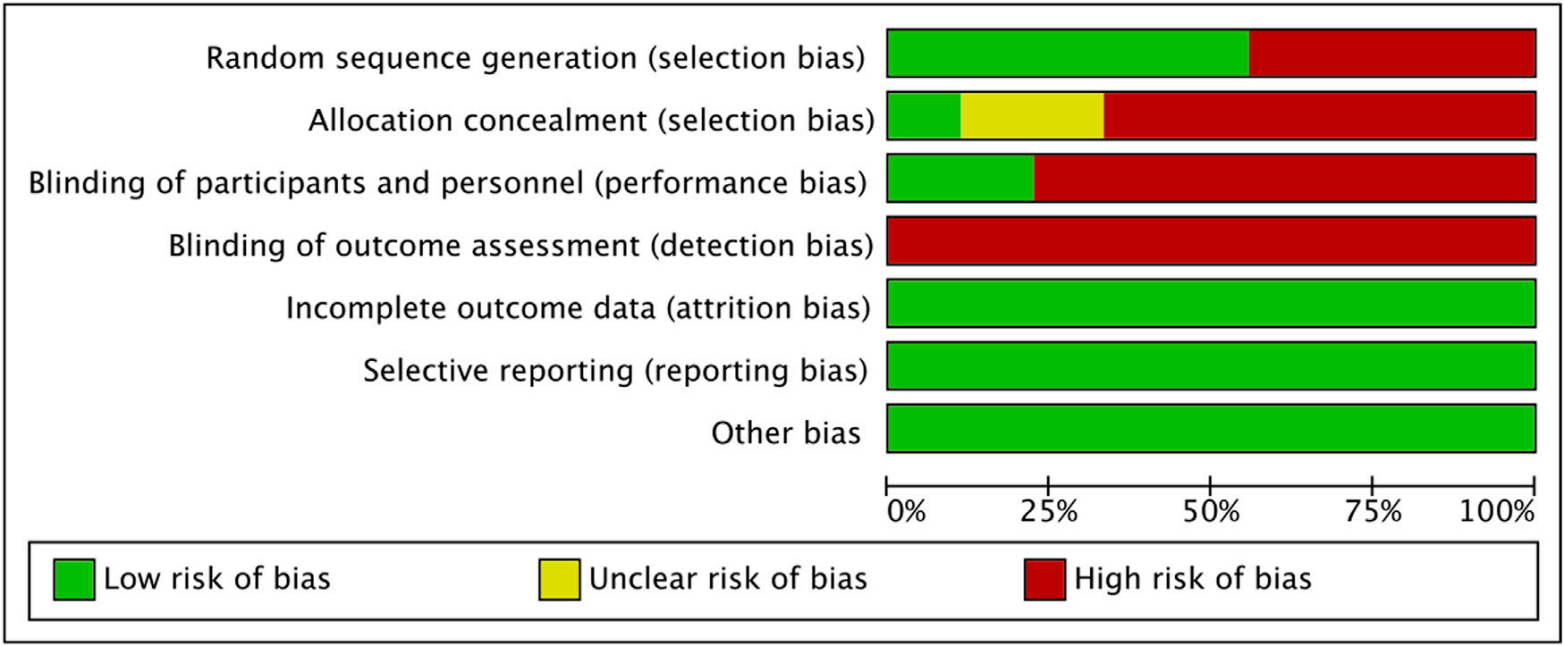
Risk of bias summary. Review authors’ judgements about each risk of bias item for each included study.

The final results were shown in the form of summary (Figure 2) and graph (Figure 3). All studies had low risks of attrition bias, reporting bias and other bias. Selection bias of random sequence generation was high in four studies (Kirsteins et al., 1991; Zhu et al., 1999; Lee and Jeong, 2006) and was low in the other five studies (Van Deusen and Harlowe, 1987; Lee, 2005; Wang, 2008; Shin et al., 2015; Liang, 2020). Selection bias of allocation concealment was high in six studies (Kirsteins et al., 1991; Zhu et al., 1999; Lee and Jeong, 2006; Shin et al., 2015; Liang, 2020), unclear in two studies (Van Deusen and Harlowe, 1987; Lee, 2005) and low in one study (Wang, 2008). Performance bias of blinding of participants and personnel was high in seven studies (Kirsteins et al., 1991; Zhu et al., 1999; Lee, 2005; Lee and Jeong, 2006; Wang, 2008; Liang, 2020) and was low in the other two studies (Van Deusen and Harlowe, 1987; Shin et al., 2015). Detection bias blinding of outcome assessment was high in all the included studies.

Regarding the risk of bias of individual studies, four trials (tow trials from one study) were considered with high risk (Kirsteins et al., 1991; Zhu et al., 1999; Lee and Jeong, 2006). In contrast, two studies were rated as medium risk (Lee, 2005; Liang, 2020) and three studies as low risk (Van Deusen and Harlowe, 1987; Wang, 2008; Shin et al., 2015).

### Outcomes and analysis

#### Time to walk 50 feet

**FIGURE 4:**
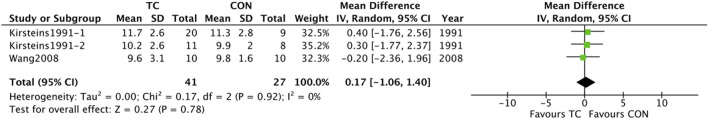
A Forest plot of the meta-analyses compared TC group with control group for changing in time to walk 50 feet.

We collected the data from three studies (Kirsteins et al., 1991; Wang, 2008) together and acquired evidence that TC therapy could not significantly improve time to walk 50 feet, with MD 0.17 (95% CI −1.06–1.40) in a random effect model (Figure 4). Tow independent CCTs were from one article (Kirsteins et al., 1991).

#### Joint tenderness

**FIGURE 5:**
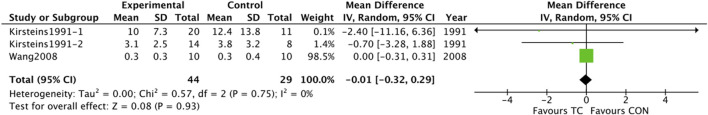
A Forest plot of the meta-analyses compared TC group with control group for changing in joint tenderness.

The data from three studies (Kirsteins et al., 1991; Wang, 2008) were collected together and evidence was acquired that TC therapy could not significantly improve joint tenderness, with MD -0.01 (95% CI −0.32 to 0.29) in a random effect model (Figure 5). Tow independent CCTs were from one article (Kirsteins et al., 1991).

#### Number of swollen joints

**FIGURE 6:**
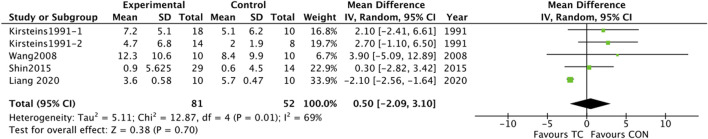
A Forest plot of the meta-analyses compared TC group with control group for changing in number of swollen joints.

The data were collected from five studies (Kirsteins et al., 1991; Wang, 2008; Shin et al., 2015; Liang, 2020) suggested that TC therapy could not significantly improve number of swollen joints, with MD 0.50 (95% CI −2.09 to 3.10) in a random effect model (Figure 6). Tow independent CCTs were from one article (Kirsteins et al., 1991).

#### Number of tender joints

**FIGURE 7:**
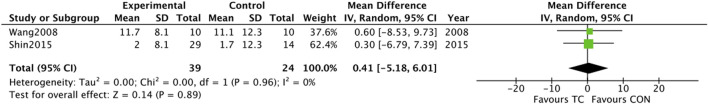
A Forest plot of the meta-analyses compared TC group with control group for changing in number of tender joints.

The data from two studies (Wang, 2008; Shin et al., 2015) together indicated that TC therapy could not significantly improve number of tender joints, with MD 0.41 (95% CI −5.18 to 6.01) in a random effect model (Figure 7).

#### Handgrip strength

**FIGURE 8:**
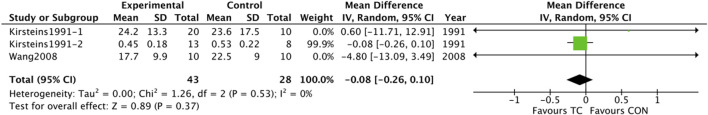
A Forest plot of the meta-analyses compared TC group with control group for changing in handgrip strength.

After the collection of the data from three studies (Kirsteins et al., 1991; Wang, 2008), the results showed that TC therapy could not significantly improve handgrip strength, with MD −0.08 (95% CI −0.26 to 0.10) in a random effect model (Figure 8). Tow independent CCTs were from one article (Kirsteins et al., 1991).

#### Pain

**FIGURE 9:**
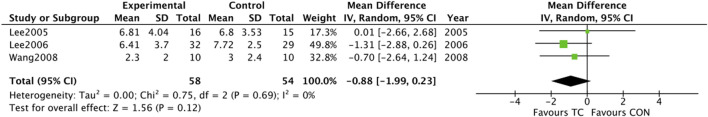
A Forest plot of the meta-analyses compared TC group with control group for changing in pain.

The data from three studies (Lee, 2005; Lee and Jeong, 2006; Wang, 2008) showed that TC therapy could not significantly improve pain, with MD −0.88 (95% CI −1.99 to 0.23) in a random effect model (Figure 9).

#### HAQ

**FIGURE 10:**
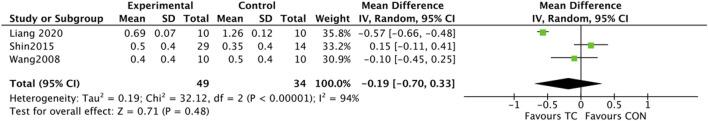
A Forest plot of the meta-analyses compared TC group with control group for changing in HAQ.

After the collection of the data from three studies (Wang, 2008; Shin et al., 2015; Liang, 2020), the results showed that TC therapy could not significantly improve HAQ, with MD −0.19 (95% CI −0.70 to 0.33) in a random effect model (Figure 10).

#### Withdrawals overall

**FIGURE 11:**
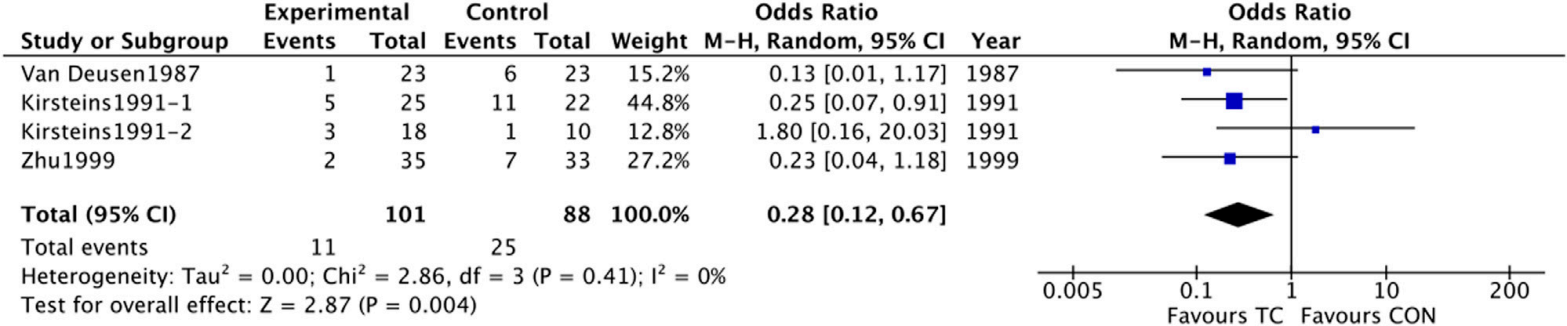
A Forest plot of the meta-analyses compared TC group with control group for changing in withdrawals overall.

The data from four studies (Van Deusen and Harlowe, 1987; Kirsteins et al., 1991; Zhu et al., 1999) was combined and provided evidence that TC therapy could significantly improve withdrawals overall during the study, with OR 0.28 (95% CI 0.12–0.67) in a random effect model (Figure 11). Tow independent CCTs were from one article (Kirsteins et al., 1991).

## Discussion

351 participants were included in this meta-analysis from nine trials. Three of them were CCTs and six were RCTs in total. All patients were diagnosed by rheumatologists or clinicians in department of rheumatology. We used the collective data to perform a meta-analysis and found that TC could significantly improve the withdrawals overall during the study. Available data suggested that TC was not linked closely with serious adverse events. However, TC cannot improve physical functions of RA patients. Additionally, the included studies were assessed as having a relative high risk of bias. Four trials with high risk might greatly reduces the credibility of the results. Two studies rated with medium risk and three studies with low risk might have relatively small impact on the confidence of the results. Therefore, the confidence in the findings were seriously reduced.

RA is a second common form of arthritis. However, treating strategy is limited and medications are frequently toxic (Nagy et al., 2022). Therefore, RA patients turn to complementary and alternative therapies often (Zhao et al., 2017). The value of regular physical activity is well documented in the management of RA (Hu et al., 2021; Roodenrijs et al., 2021). Physical activity for patients with RA needs to be sustainable and enjoyable, however most of them have less physically active than the general population in fact (Hu et al., 2021). In addition, A systematic review about efficacy of occupational therapy-related interventions for adults with RA concluded strong evidence to support the use of aerobic exercise, such as TC (Siegel et al., 2017).

Recently, TC has been applied with substantial benefits in patients with RA. Intensity in TC is low and equivalent to walking 6 km/h and produces a secondary increase in heart rate (Jin, 1992), which comprised rhythmic movements and emphasis on body balance and coordination (Song et al., 2010). There are different kinds of actions, such as bend knees slightly, keep arms below the shoulder level, forward or backward strides, and turn around while shifting the center of gravity (Song et al., 2007). Although TC has lots of styles and flexible action details, it can be assumed that the major function of TC is similar. TC is considered safe in patients with RA, especially long-standing and dramatically physically inactive individuals (Kirsteins et al., 1991). This is the same with the withdraw overall outcome in our meta-analysis. TC could decrease the percentage of dropouts in trials.

Studies had demonstrated a favorable effect or tendency to improve physical function (Chen et al., 2016). A study indicated that the positive effects of TC were attributed to increases in the muscle strength and endurance of the lower extremity (Song et al., 2010). It may also help to improve body balance and stabilize the weighted joints thereby reducing the risk of falling (Wang, 2009). Additionally, another review about TC treating RA concluded that there were positive effects on a selected range of motion outcomes (Han et al., 2004). However, investigators thought that TC had no effectiveness of TC treating RA in another meta-analysis (Lee et al., 2007). Our results also showed TC cannot improve physical function of RA patients.

The primary limitation of this review is the small total number of eligible trials. Therefore, the results of the studies might or might not apply to the majority of RA patients; there were not enough studies for conclusive judgment, especially the side effects of TC. TC only could be assumed with a low risk of injury as a treatment method. In addition, we tried our best to search relevant articles in different ways, but we could not make sure that all the relevant studies were included. So, the bias from selecting the studies for inclusion in a meta-analysis could not be avoided.

## Conclusion

The results of our systematic review and meta-analysis have provided the newest evidence on TC for the treatment of RA. It suggests that TC is a safe method to exercise for RA patients as the lower withdrawals overall. However, TC cannot improve physical function of RA patients. In addition, as the high risk of bias of included studies, the confidence in the findings was seriously reduced. More high-quality clinical studies are needed to further update the results.

## Data Availability

The original contributions presented in the study are included in the article/Supplementary Material, further inquiries can be directed to the corresponding authors.

## Abbreviations

CCTs, controlled clinical trials; CIs, confidence intervals; HAQ, health assessment questionnaire; MD, mean difference; OR, odds ratio; RA, rheumatoid arthritis; RCTs, randomized controlled trials; TC, Tai Chi.
