# Corrigendum: The effects of Tai Chi on physical function and safety in patients with rheumatoid arthritis: a systematic review and meta-analysis

**DOI:** 10.3389/fphys.2023.1260536

**Published:** 2023-08-04

**Authors:** Haiyang Wu, Qiang Wang, Guowei Wen, Junhao Wu, Yiru Wang

**Affiliations:** ^1^ Huangpu Branch, Shanghai Ninth People’s Hospital, Shanghai Jiao Tong University School of Medicine, Shanghai, China; ^2^ Shanghai Traditional Chinese Medicine (TCM), Integrated Hospital, Shanghai University of Traditional Chinese Medicine, Shanghai, China; ^3^ Longhua Hospital, Shanghai University of Traditional Chinese Medicine, Shanghai, China

**Keywords:** physical exercise, arthritis, pain, joint tenderness, swollen joints, health assessment questionnaire

In the published article, there was an error in **Affiliation** 1. Instead of “Huangpu Branch, Shanghai Ninth People’s Hospital affiliated to Shanghai Jiaotong University, Shanghai, China”, it should be “Huangpu Branch, Shanghai Ninth People’s Hospital, Shanghai Jiao Tong University School of Medicine, Shanghai, China”.

The authors apologize for this error and state that this does not change the scientific conclusions of the article in any way. The original article has been updated.

