# Supplementary material for: The effects of Tai Chi on physical function and safety in patients with rheumatoid arthritis: A systematic review and meta-analysis
Source: Front Physiol. 2023 Jan 26;14:1079841. doi: 10.3389/fphys.2023.1079841 (PMC9909273; doi:10.3389/fphys.2023.1079841)
Supplement: Supplementary file 1 [file DataSheet1.pdf]

Search strategy in Pubmed database

1 Rheumatoid Arthritis [mh]

2 Juvenile Arthritis [mh]

3 (Juvenile Arthritis or Arthritis, Juvenile Idiopathic or Idiopathic Arthritis, Juvenile or Arthritis, Juvenile Rheumatoid or Rheumatoid Arthritis, Juvenile or Enthesitis-Related Arthritis, Juvenile or Arthritis, Juvenile Enthesitis-Related or Enthesitis Related Arthritis, Juvenile or Juvenile Enthesitis- Related Arthritis or Juvenile Chronic Arthritis or Juvenile Idiopathic Arthritis or Polyarthritis, Juvenile, Rheumatoid Factor Negative or Polyarthritis, Juvenile, Rheumatoid Factor Positive or Psoriatic Arthritis, Juvenile or Arthritis, Juvenile Psoriatic or Juvenile Psoriatic Arthritis or Systemic Arthritis, Juvenile or Arthritis, Juvenile Systemic or Juvenile Systemic Arthritis or Arthritis, Juvenile Chronic or Chronic Arthritis, Juvenile or Juvenile Rheumatoid Arthritis) [tw]

4 2 or 3

5 Felty Syndrome [mh]

6 (Rheumatoid Arthritis or Splenomegaly and Neutropenia) [tw]

7 5 or 6

8 Still's Disease, Adult-Onset [mh]

9 (Still's Disease, Adult Onset or Stills Disease, Adult-Onset or Adult-Onset Still's Disease or Adult Onset Still's Disease or Adult-Onset Stills Disease or Still Disease, Adult-Onset or Still Disease, Adult Onset or Adult-Onset Still Disease or Adult Onset Still Disease) [tw]

10 8 or 9

11 1 or 4 or 7 or 10

12 Tai Chi [mh]

13 Tai Ji [mh]

14 (Tai-ji or Tai Chi or Chi, Tai or Tai Ji Quan or Ji Quan, Tai or Quan, Tai Ji or T'ai Chi or Tai Chi Chuan) [tw]

15 13 or 14

16 Dance Therapy [mh]

17 (Therapy, Dance or Dance Therapies or Therapies, Dance) [tw]

18 16 or 17

19 12 or 15 or 18

20 randomized controlled trial [pt]

21 controlled clinical trial [pt]

22 clinical trial [tiab]

23 human trials as topic [mesh: noexp]

24 trial [ti]

25 20 or 21 or 22 or 23 or 24

26 humans [mh] NOT animals [mh]

27 25 and 26

28 11 and 19 and 27

Pubmed search syntax

[mh] denotes a Medical Subject Heading (Mesh) term (‘exploded’);

[tw] denotes text word;

[pt] denotes a Publication Type term;

[tiab] denotes a word in the title or abstract; [sh] denotes a subheading;

[mesh: noexp] denotes a Medical Subject Heading (Mesh) term (not ‘exploded’);

[ti] denotes a word in the title.

Search strategy for Cochrane Library database

#1 MeSH descriptor: [Rheumatoid Arthritis] explode all trees

#2 MeSH descriptor: [Arthritis] explode all trees

#3 (Juvenile Arthritis):ti,ab,kw

#4 (Idiopathic Arthritis):ti,ab,kw

#5 (Enthesitis-Related Arthritis):ti,ab,kw

#6 (Juvenile Idiopathic Arthritis):ti,ab,kw

#7 (Polyarthritis):ti,ab,kw

#8 (Chronic Arthritis):ti,ab,kw

#9 #1 or #2 or #3 or #4 or #5 or #6 or #7 or #8

#10 MeSH descriptor: [Tai Chi] explode all trees

#11 MeSH descriptor: [Taiji] explode all trees

#12 #10 or #11

#13 #9 and #12

Cochrane Library search syntax

[ti] denotes a word in the title.

[ab] denotes a word in the abstract.

[kw] denotes a word in the keywords.

Search strategy for EMBASE

#1 ‘Rheumatoid Arthritis’ /exp

#2 ‘Arthritis’ /exp

#3 ‘Juvenile Arthritis’:ab,ti

#4 ‘Idiopathic Arthritis’:ab,ti

#5 ‘Enthesitis-Related Arthritis’:ab,ti

#6 ‘Juvenile Idiopathic Arthritis’:ab,ti

#7 ‘Polyarthritis’:ab,ti

#8 ‘Chronic Arthritis’:ab,ti

#9 #1 OR #2 OR #3 OR #4 OR #5 OR #6 OR #7 OR #8

#10 ‘Tai Chi’ /exp

#11 ‘Taiji’ /exp

#12 #10 or #11

#13 ‘controlled clinical trial’ /exp

#14 #9 and #12 and #13

EMBASE search syntax

[exp] denotes explosion.

[ab] denotes a word in the abstract.

[ti] denotes a word in the article title.

Search Strategy for Web of science:

#1 TS= (Tai Chi)

#2 TS= (Taiji)

#3 #1 AND #2

#4 TS= Rheumatoid Arthritis

#5 TS= (Arthritis OR Juvenile Arthritis OR Idiopathic Arthritis OR Enthesitis-Related Arthritis OR Juvenile Idiopathic Arthritis OR Polyarthritis OR Chronic Arthritis)

#6 #4 AND #5

#7 #3 AND #6

#8 TS= (Controlled Clinical Trials OR trial OR placebo OR groups OR control OR controlled)

#9 #7 and #8

Search Strategy for China National Knowledge Infrastructure

FT=对照+对照研究+对照试验 AND SU=太极+太极拳 AND SU=类风湿性关节炎+类风湿关节炎+关节炎+类风湿+RA
